# Smokers or non-smokers: who benefits more from immune checkpoint inhibitors in treatment of malignancies? An up-to-date meta-analysis

**DOI:** 10.1186/s12957-020-1792-4

**Published:** 2020-01-20

**Authors:** Jiahang Mo, Xiao Hu, Lihu Gu, Bangsheng Chen, Parikshit Asutosh Khadaroo, Zefeng Shen, Lei Dong, Yuqi Lv, Marylin Nyaradzo Chitumba, Jiequan Liu

**Affiliations:** 1 The Second Clinical Medical College, Zhejiang Chinese Medical University, Zhejiang, Hangzhou China; 2 School of Stomatology, Zhejiang Chinese Medical University, Zhejiang, Hangzhou China; 3 Department of General Surgery, HwaMei Hospital, University of Chinese Academy of Sciences, Ningbo, Zhejiang, China; 4 Emergency Medical Center, The Second Hospital of Yinzhou, 998 North Qianhe Road, Yinzhou District, Ningbo, Zhejiang, 315100 China; 5 Monash University School of Medicine, Nursing and Health Sciences, Melbourne, Australia; 6 Department of Pneumology, The Second Affiliated Hospital of Zhejiang Chinese Medical University, Zhejiang, Hangzhou China; 7 International Education College, Zhejiang Chinese Medical University, Zhejiang, Hangzhou China

**Keywords:** Smoking, Immunotherapy, Immune checkpoint inhibitors, Anti-PD-1/PD-L1, Meta-analysis

## Abstract

**Background:**

Immune checkpoint inhibitors, which are a milestone in anti-cancer therapy, have been applied in the treatment of multiple malignancies. Real-world data have suggested that smoking status may be associated with the efficacy of anti-PD-1/PD-L1 therapy. Hereby, to evaluate “smoking benefit or not”, we included numerous high-quality randomized controlled clinical trials (RCTs) without any restriction on category.

**Methods:**

A systematic search of online database was performed from July 2010 to July 2019. Eligible studies included phase II/III RCTs comparing PD-1/PD-L1 inhibitors with chemotherapy in the treatment of multiple carcinomas and contained subgroup analysis of smoking status. Then, related hazard ratios (HRs) with 95% confidence intervals (CIs) of overall survival (OS) were pooled.

**Results:**

In the initial meta-analysis, compared with chemotherapy, the OS of non-smokers (HR, 0.81; 95% CI, 0.67–0.98) and smokers (HR, 0.77; 95% CI, 0.71–0.83) were significantly prolonged with PD-1/PD-L1 inhibitors. Outcomes from subgroup analysis showed that in anti-PD-1/PD-L1 monotherapy groups, non-smokers showed no significant improvement in OS (HR, 0.94; 95% CI, 0.83–1.06), while the OS of smokers was significantly prolonged (HR, 0.79; 95% CI, 0.74–0.85); in groups of PD-1/PD-L1 inhibitors combined with chemotherapy, the OS of non-smokers (HR, 0.45; 95% CI, 0.28–0.71) and smokers (HR, 0.72; 95% CI, 0.61–0.85) were significantly prolonged. Combined ipilimumab and chemotherapy showed no significance in both groups.

**Conclusion:**

Smokers benefit from either anti-PD-1/PD-L1 monotherapy or the combined regimen compared with chemotherapy. Considering cost-effectiveness, monotherapy was recommended to smokers. For non-smokers, only the combined regimen was feasible in non-small cell lung cancer.

## Introduction

Immune checkpoint inhibitors (ICIs), including PD-1/PD-L1 and CTLA-4 inhibitors, are monoclonal antibodies that remove tumor cells by activating T lymphocytes and enhancing immune response [1]. In 2010, a phase III randomized controlled clinical trial (RCT) [2] confirmed that ipilimumab, a CTLA-4 inhibitor, significantly improved overall survival (OS) in patients with metastatic melanoma compared with traditional vaccine therapy. Soon after, ipilimumab became the first FDA-approved ICI in 2011. The success of CTLA-4 inhibitors greatly stimulated the research of PD-1/PD-L1 inhibitors. Subsequently, trials with CheckMate057 [3], CheckMate141 [4], and KEYNOTE 045 [5] demonstrated significant efficacy of PD-1 inhibitors (including nivolumab and pembrolizumab) in multiple carcinomas. In the process of advancing human anti-cancer treatment, ICIs are applied to treat multiple malignancies and are replacing the standard therapy.

However, real-world data had shown that even with the same treatment, due to individual characteristics, the therapeutic effect can be quite different among cancer patients with the same indication. Meanwhile, the high cost of checkpoint inhibitors greatly increases the financial burden on patients [6]. Therefore, researchers have conducted a large number of studies to explore factors affecting the efficacy of checkpoint inhibitors, aiming to provide a solid foundation in the selection of clinical treatment regimens. Currently, wide research has confirmed that PD-L1 expression in tumor tissues can be used to predict the efficacy of anti-PD-1/PD-L1 therapy [5, 7, 8]. Similarly, tumor mutation burden (TMB) is considered to be a robust predictor of efficacy in immunotherapy of non-small cell lung cancer (NSCLC) [9]. In terms of gender, sex-related dimorphism in immune system response is acknowledged. To identify the general perception in immunotherapy, a high-quality meta-analysis conducted by Conforti et al. [10] also pointed out that males benefit more than females in the treatment using ICIs. In addition, the benefit of anti-PD-1/PD-L1 therapy varies by tumor type. In melanomas, Ribas et al. [11] found that intratumoral injection of an oncolytic virus will enhance the immune recognition of cancer, resulting in a high response rate in patients with advanced disease. In squamous cell carcinoma of head and neck (HNSCC), higher expression of the immunotherapy target PD-1 in HPV+ immune cells compared to HPV− cells was observed, suggesting that HPV+ patients may preferentially benefit from anti-PD-1 therapy [12]. In NSCLC, the benefit of anti-PD-1/PD-L1 therapy was even correlated with intestinal flora [13]. Studies on the relevant mechanisms are also rife in the field of tumor molecular biology. Hugo et al. [14] analyzed somatic mutations and transcriptome of melanoma and concluded that the anti-PD-1 response might be improved by weakening the biological process of IPRES, a transcriptional signature related to innate anti-PD-1 resistance. Segovia et al. [15] found that TMEM176B inhibitor (BayK8644) can promote CD8+ T cell-mediated tumor suppression and enhance anti-tumor activities of anti-CTLA-4 and anti-PD-1 antibodies. For severe glycosylation of PD-L1, Lee et al. [16] proposed to remove glycosylated N-chain, further improved the detection of PD-L1, and predicted the therapeutic effect of anti-PD-1/PD-L1.

Smoking is considered an adverse behavior and has been implicated in many clinical studies of anti-PD-1/PD-L1 therapy as a part of patient characteristics. Previously, two pieces of meta-analyses have indicated that in NSCLC, smokers have a benefit tendency in anti-PD-1 therapy, while non-smokers may not [17, 18]. However, another meta-analysis published in *JAMA Oncology* found the opposite [19]. Coincidentally, a number of articles published recently also suggested this opposite result [20, 21]. To investigate these conflicts, we included a large number of high-quality RCTs without any restriction on carcinoma category to evaluate “smoking benefit or not” and provide some reliable evidence when choosing therapy regimens.

## Methods

### Literature search

This meta-analysis was conducted in line with the Preferred Reporting Items for Systematic Reviews and Meta-Analyses guidelines [22]. We searched PubMed, Embase, Web of Science, and the Cochrane Library from 31 July 2010 until 31 July 2019 for relevant articles. The searched terms consisted of three parts. [Neoplasms]: “Neoplasms” was selected in the MeSH term and “Tumor”, “Cancer”, “Carcinoma”, “Malignancy”, “Malignant neoplasms” were retrieved in the field of Title/Abstract. All the above were connected by “OR”. [Immune-checkpoint inhibitors]: “Immune-checkpoint inhibitor”, “PD-1”, “PD-L1”, “Pembrolizumab”, “Nivolumab”, “Atezolizumab”, “Avelumab”, “Durvalumab”, “CTLA-4”, “Ipilimumab” and “Tremelimumab”, were retrieved in all fields, then connected by “OR”. [Randomized controlled trial]: “Randomized controlled trial”, “clinical trial” were selected in the MeSH term to restrict literature types. “AND” was then used to connect these parts and used to get the results we needed. Without omitting the negative results, we did not restrict search terms related to smoking. The reference lists of retrieved studies and relevant reviews were also searched to identify additional eligible studies missed by the search strategies, and the process was performed repeatedly until no further article was found. Two investigators performed the reference search independently; when divergences appeared, a third investigator was consulted.

### Study eligibility

The inclusion criteria were randomized controlled trials of ICIs versus standard therapy, phase II or III and that baseline profile of patients included items such as smoking status or tobacco use. Exclusion criteria were republished, non-randomized controlled trials, no OS of non-smokers and smokers on its subgroup analysis and no chemotherapy control arm. If more than one publication was found for the same trial, the most complete and updated version was included in the final analysis. Following identification of target objects, Cochrane collaboration’s tool for assessing risk of bias was used to assess the quality of included studies [23].

### Data extraction

Data was collected independently by two investigators (Mo and Hu). Discrepancies were consulted by a third investigator (Gu). All data was extracted from primary publications and their associated online appendices and were collected using a pre-designed electronic form. The following information was involved: first author’s name, year of publication, trial phase, carcinoma category, therapy line, feature of each study, regimen of experimental and control arms, number of evaluable patients, and the percentages and hazard ratios (HRs) for OS (with the relative 95% CI) of non-smokers and smokers on each study.

### Statistical analysis

All statistical analyses were conducted with StataSE 12.0. *P* value less than 0.05 was considered statistically significant. Some studies divided smoking status into three categories: never smoking, former smoking, and current smoking. For convenience of definition, we combined the HRs of OS of former smoking and current smoking and finally renamed it “smoker”. Therefore, summary estimate was calculated using random or fixed-effects models according to heterogeneity. Heterogeneity among studies was tested using Cochran Chi-square test and *I*^2^, when *I*^2^ > 50%, and a random-effects model was chosen to pool the outcomes, while a fixed-effects model was used when *I*^2^ < 50%. Publication bias and sensitivity analysis were performed depending on the number of studies included in each meta-analysis.

## Results

### Literature search

**Fig. 1:**
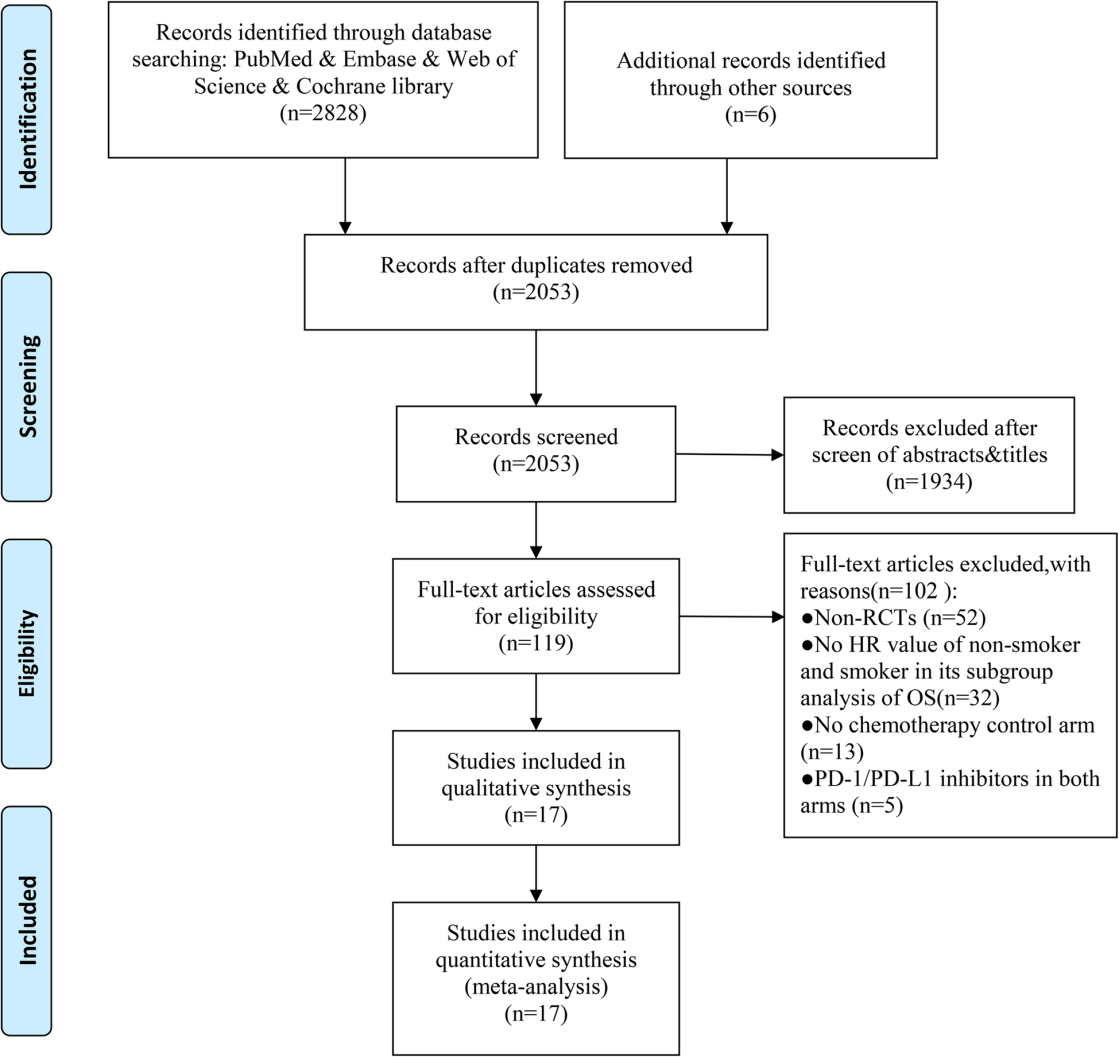
Flowchart of literature screening process

According to the search strategies from Section 2.1, 2828 citations were obtained from PubMed, Embase, Web of Science, and the Cochrane library database. Six publications were supplied by manually searching the reference lists and reviewed articles. After removal of duplicates, 2053 records remained in total. All titles and abstracts were screened and 1934 publications were excluded. After more detailed evaluation, 119 articles were submitted; of all the remaining manuscripts, 102 were excluded according to the following criteria breakdown: 52 studies were non-RCTs, 32 studies lack OS of non-smokers and smokers in their subgroup analysis (with the relative 95% CI), 13 studies were lack of chemotherapy control arms, and 5 studies contained PD-1/PD-L1 in both arms. Eventually, 17 RCTs were included in the meta-analysis [24–31] and a total of 11790 patients involved. Figure 1 shows the flow chart of the selection process and detailed identification.

### Study characteristics

The characteristics and baseline of the included studies are summarized in Table 1. Among the 17 studies, 16 studies were in phase III RCTs except one in phase II. There were 3 types of carcinomas in this meta-analysis, including 13 studies of lung cancer, 2 studies of urothelial carcinoma (UC) and 2 studies of HNSCC. For convenience, the latter two were defined as “other cancer”. In these studies, subgroup analysis of non-smokers and smokers were included, and the HRs of OS of two groups (with the relative 95% CI) were served as the only index in this article. However, because the Brahmer et al. study [32] had an extremely low proportion of non-smokers, these data were not available.

**Table 1:** Main characteristics of included studies

Author	Year	Phase	Category	Therapy line	Feature	Exp arm (Pts *N*)	Ctr arm (Pts *N*)	Population	OS	
									HR	95% CI
Borghaei et al.	2015	III	NSCLC	> 1	Non-squamous	Nivolumab (292)	Docetaxel (290)	Non-smoker (20%)	1.02	0.64–1.16
								Smoker (79%)	0.70	0.56–0.86
Brahmer et al	2015	III	NSCLC	> 1	Squamous	Nivolumab (135)	Docetaxel (137)	Non-smoker (6%)	NA	NA
								Smoker (92%)	0.59	0.44–0.80
Fehrenbacher et al.	2016	II	NSCLC	> 1	Previously treated	Atezolizumab (144)	Docetaxel (143)	Non-smoker (20%)	0.55	0.24–1.25
								Smoker (80%)	0.75	0.54–1.04
Rittmeyer et al.	2017	III	NSCLC	> 1	Previously treated	Atezolizumab (613)	Docetaxel (612)	Non-smoker (17%)	0.91	0.65–1.29
								Smoker (83%)	0.78	0.67–0.90
Carbone et al.	2017	III	NSCLC	1	Stage IV	Nivolumab (271)	Chemotherapy (270)	Non-smoker (11%)	1.02	0.54–1.93
								Smoker (89%)	1.08	0.86–1.37
Barlesi et al.	2018	III	NSCLC	> 1	Platinum-treated advanced	Avelumab (396)	Docetaxel (396)	Non-smoker (17%)	1.69	0.97–2.95
								Smoker (83%)	0.83	0.66–1.04
Mok et al.	2019	III	NSCLC	1	PD-L1 positive	Pembrolizumab (636)	Chemotherapy (615)	Non-smoker (22%)	1.00	0.73–1.37
								Smoker (78%)	0.80	0.60–1.06
Gandhi et al.	2018	III	NSCLC	1	Metastatic	Pembrolizumab+chemotherapy (410)	Placebo+chemotherapy (206)	Non-smoker (12%)	0.23	0.10–0.54
								Smoker (88%)	0.54	0.41–0.71
Antonia et al.	2018	III	NSCLC	> 1	Stage III	Durvalumab+chemoradiotherapy (476)	Placebo+chemoradiotherapy (237)	Non-smoker (9%)	0.35	0.16–0.76
								Smoker (91%)	0.72	0.56–0.92
West et al.	2019	III	NSCLC	1	Non-squamous	Atezolizumab+chemotherapy (451)	Chemotherapy (228)	Non-smoker (10%)	0.55	0.26–1.19
								Smoker (90%)	0.81	0.65–1.02
Reck et al.	2019	III	NSCLC	> 1	Chemotherapy-naive metastatic	ABCP (400)	BCP (400)	Non-smoker (20%)	0.66	0.41–1.05
								Smoker (80%)	0.80	0.65–0.89
Bellmunt et al.	2017	III	UC	> 1	Advanced second line	Pembrolizumab (270)	Chemotherapy (272)	Non-smoker (35%)	1.06	0.72–1.55
								Smoker (65%)	0.52	0.24–1.11
Powles et al.	2018	III	UC	> 1	Advanced or metastatic	Atezolizumab (467)	Chemotherapy (464)	Non-smoker (28%)	0.80	0.60–1.06
								Smoker (72%)	0.87	0.72–1.04
Ferris et al.	2016	III	HNSCC	> 1	Recurrent	Nivolumab (240)	Standard therapy (121)	Non-smoker (19%)	0.58	0.32–1.06
								Smoker (77%)	0.71	0.52–0.99
Cohen et al.	2019	III	HNSCC	> 1	Recurrent or metastatic	Pembrolizumab (247)	Standard therapy (248)	Non-smoker (27%)	0.90	0.60–1.35
								Smoker (73%)	0.77	0.60–0.98
Reck et al.	2016	III	SCLC	1	Extensive-stage	Ipilimumab+chemotherapy (478)	Placebo+chemotherapy (476)	Light smoker (36%)	1.02	0.80–1.30
								Heave smoker (57%)	1.09	0.89–1.32
Govindan et al.	2017	III	NSCLC	1	Squamous	Ipilimumab+chemotherapy (388)	Placebo+chemotherapy (361)	Light smoker (12%)	1.19	0.71–1.99
								Heave smoker (88%)	0.88	0.73–1.05

*OS* overall survival, *CI* confidence interval, *HR* hazard ratio, *NA* not available, *PD-L1* programmed death ligand 1, *Pts* patients, *Exp* experimental, *Ctr* control, *Non-smoker* patients who never smoke, *Smoker* patients who ever/currently smoke, *NSCLC* non-small cell lung cancer, *UC* urothelial carcinoma, *HNSCC* head-and-neck squamous cell carcinoma, *SCLC* small cell lung cancer, *ABCP* atezolizumab plus bevacizumab plus carboplatin plus paclitaxel, *BCP* bevacizumab plus carboplatin plus paclitaxel; *Standard Therapy* standard single-agent systemic therapy (methotrexate, docetaxel, or cetuximab)

### Quality of the included studies

Cochrane collaboration’s tool for assessing risk of bias was used to assess the quality of included studies [23]. Most of the studies had a high risk of performance bias due to their open-label design (Additional file 6: Table S1). Based on high-quality RCTs, other dimensions were ensured at relatively low risk. The overall quality met the requirements of meta-analysis.

### Non-smoker vs. smoker in Anti-PD-1/PD-L1 Therapy

**Fig. 2:**
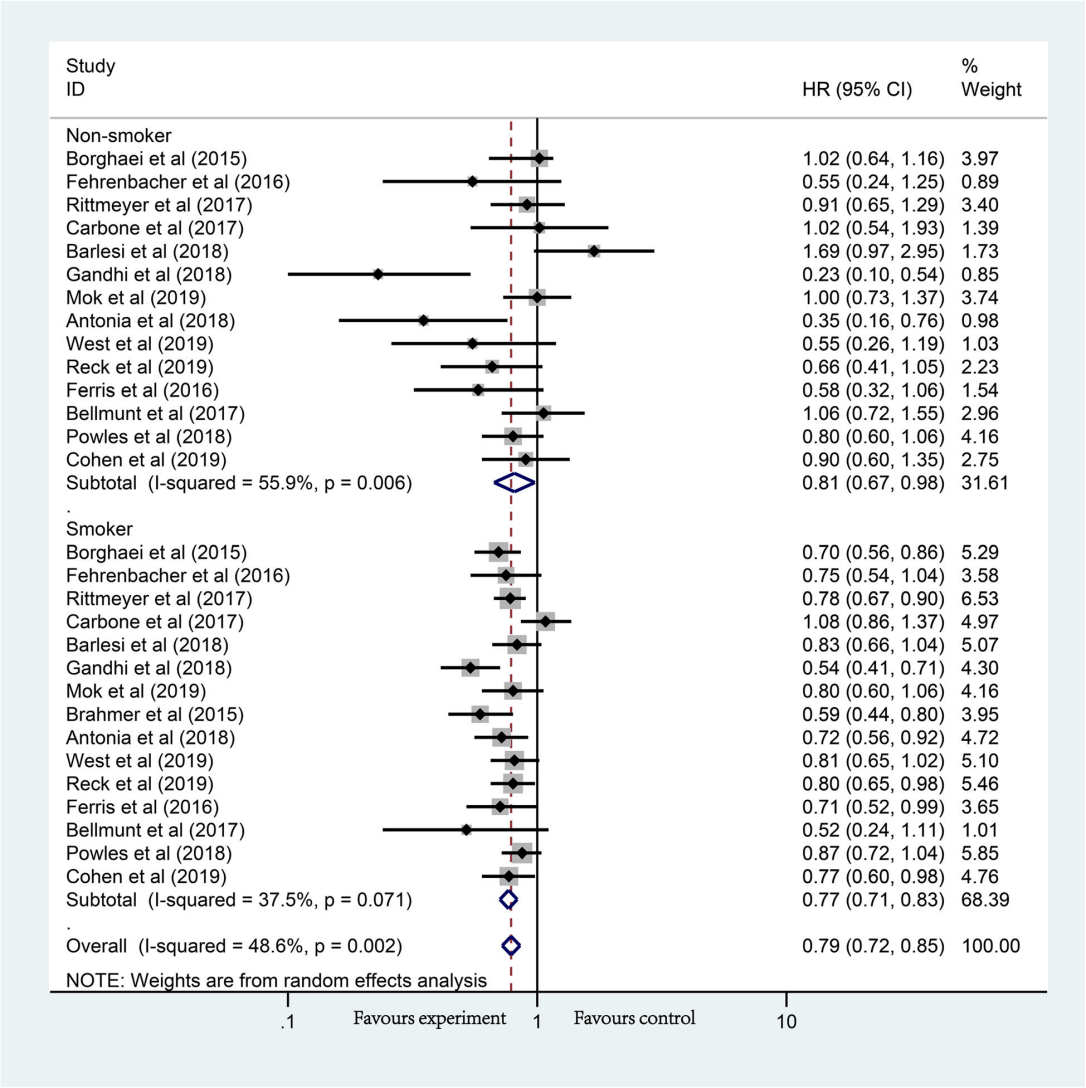
Forest plot of the long-term prognostic outcomes of anti-PD-1/PD-L1 therapy (non-smoker vs. smoker), *P*_Non-smoker_ = 0.029, *P*_Smoker_ < 0.001

Overall, we divided the population of each study into 2 fixed groups according to smoking status for meta-analysis. Compared with chemotherapy, the OS of non-smokers (HR, 0.81; 95% CI, 0.67–0.98; *P* = 0.029) and smokers (HR, 0.77; 95% CI, 0.71–0.83; *P* < 0.01) were significantly prolonged in 15 studies using PD-1/PD-L1 inhibitors (Fig. 2). Heterogeneity tests were done and suggested that there was still some heterogeneity in the total group (non-smoker fixed-group, *I*^2^ = 55.9%; smoker fixed-group, *I*^2^ = 37.5%; total, *I*^2^ = 48.6%). Because 15 studies and a total of 29 single items were under consideration here, publication bias analysis was conducted **(**Additional file 1: Figure S1) and the Egger test indicated that there is no potential publication bias in the above data (*P* = 0.203). Subsequently, sensitivity analyses have confirmed the robustness of the results (Additional file 2: Figure S2).

### Subgroup analysis

**Fig. 3:**
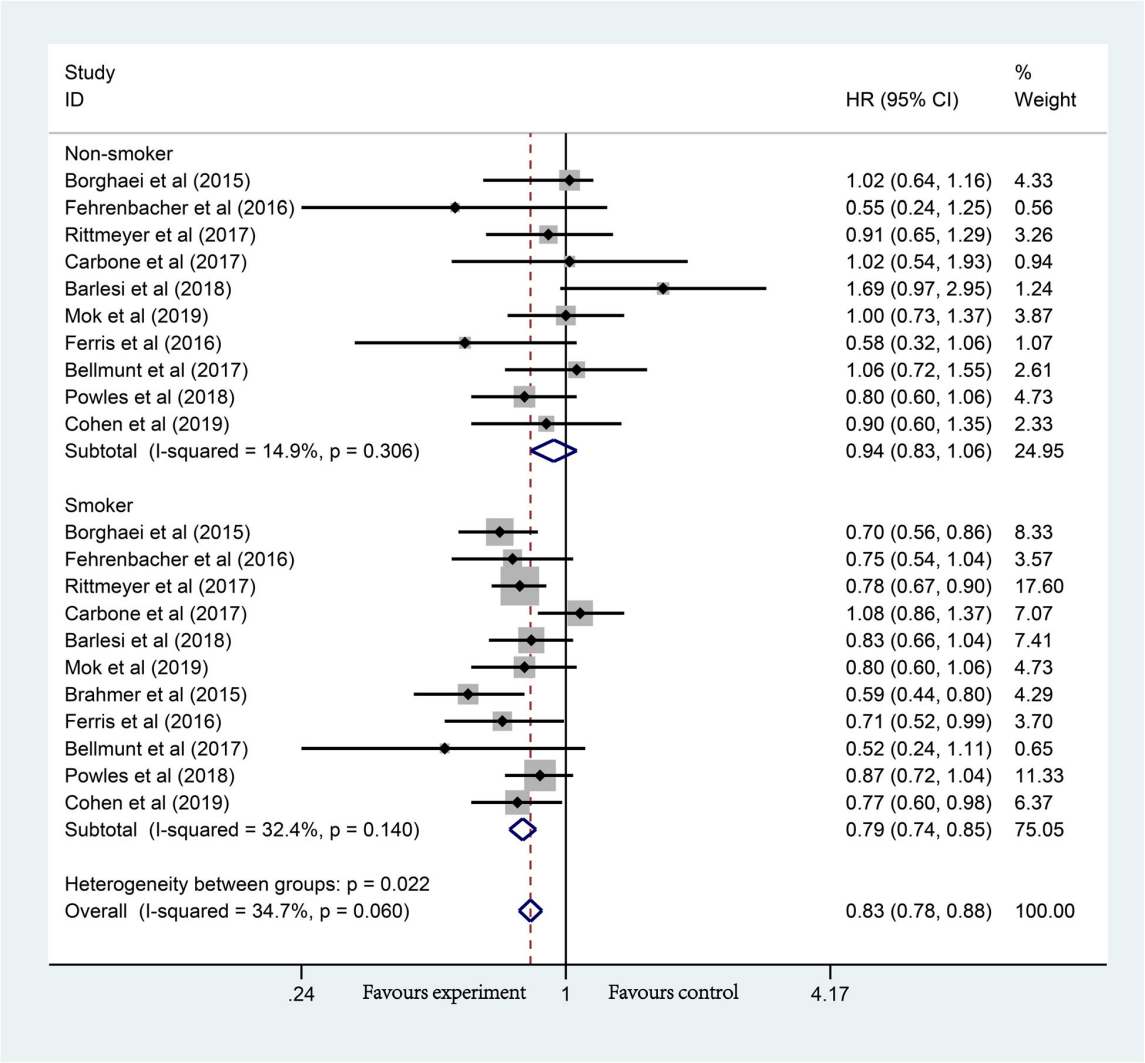
Forest plot of the long-term prognostic outcomes of anti-PD-1/PD-L1 monotherapy (non-smoker vs. smoker), *P*_Non-smoker_ = 0.304, *P*_Smoker_ < 0.001

Given the heterogeneity of anti-PD-1/PD-L1, subgroup analysis was conducted based on the therapeutic regimen. In anti-PD-1/PD-L1 monotherapy groups, non-smokers showed no significant improvement in OS compared with chemotherapy (HR, 0.94; 95% CI, 0.83–1.06; *P* = 0.304), while the OS of smokers were significantly prolonged (HR, 0.79; 95% CI, 0.74–0.85; *P* < 0.01) (Fig. 3). On this basis, carcinoma types were analyzed. In NSCLC, non-smokers showed no significant improvement in OS (HR, 1.01; 95% CI, 0.84–1.21; *P* = 0.921), while the OS of smokers were significantly prolonged (HR, 0.79; 95% CI, 0.69–0.89; *P* < 0.01) (Additional file 3: Figure S3). In other cancer, non-smokers showed no significant improvement in OS (HR, 0.85; 95% CI, 0.70–1.03; *P* = 0.094), the OS of smokers was significantly prolonged (HR, 0.80; 95% CI, 0.70–0.91; *P* = 0.001) (Additional file 4: Figure S4). The three sets of data showed an excellent consistency.

**Fig. 4:**
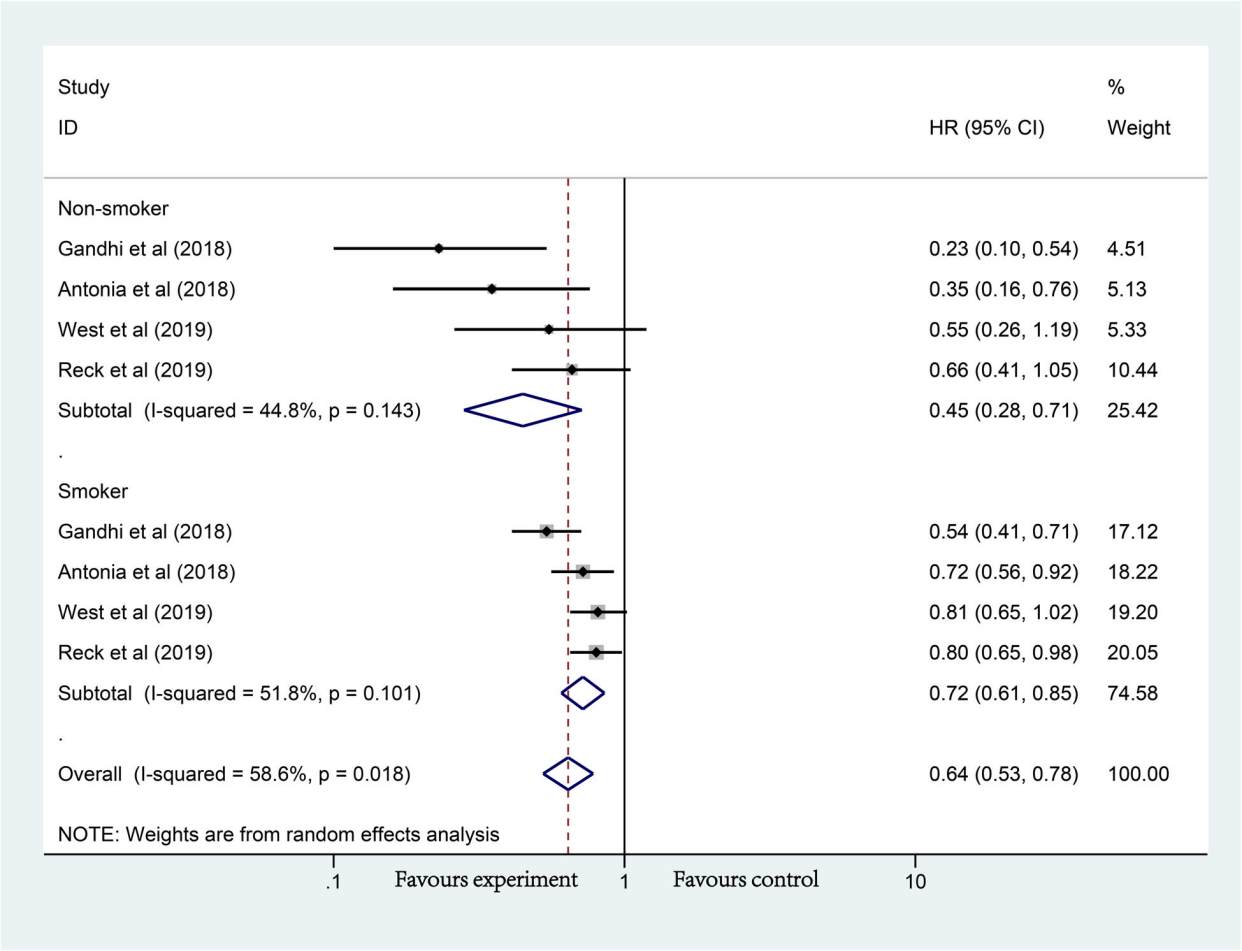
Forest plot of the long-term prognostic outcomes of the combined regimen (Anti-PD-1/PD-L1+chemotherapy) (non-smoker vs. smoker), *P*_Non-smoker_ = 0.001, *P*_Smoker_ < 0.001

In the rest of these studies, PD-1/PD-L1 inhibitors combined with chemotherapy were served as the experimental arms (the Antonia et al. [24] study was sequential therapy with PD-1/PD-L1 inhibitors after chemoradiotherapy), limited to NSCLC. In these groups, compared with chemotherapy alone, the OS of non-smokers (HR, 0.45; 95% CI, 0.28–0.71; *P* < 0.01) and smokers (HR, 0.72; 95% CI, 0.61–0.85; *P* < 0.01) was significantly prolonged (Fig. 4). Compared with smokers, non-smokers seemed to benefit more.

### Non-smoker vs. smoker in anti-CTLA-4 therapy

In view of the differences in intervention and smoking status, two ipilimumab-related studies were analyzed separately. The results showed that compared with chemotherapy alone, the OS of light smokers (HR, 1.05; 95% CI, 0.84–1.31; *P* = 0.67) and heavy smokers (HR, 0.98; 95% CI, 0.79–1.20; *P* = 0.82) were not significantly improved when combined with ipilimumab (Additional file 5: Figure S5).

## Discussion

Although previous meta-analyses have attempted to clarify the relationship between smoking and the efficacy of ICIs [17, 18], due to the limitations of the number of studies, the types of carcinomas and lack of subgroup analysis, the results remained to be confirmed. In this meta-analysis, we included a large amount of high-quality trials without any restriction on carcinoma category. On this basis, reasonable subgroup analysis allowed us to find some promising results while reducing the heterogeneity.

In the analysis of the relationship between smoking status and the efficacy of anti-PD-1/PD-L1 therapy, a meta-analysis was performed including 15 related studies (except two ipilimumab-related). Then, we drew a conclusion that regardless of smoking or not, patients in the experimental arms always benefit, which is consistent with the analysis of Lee et al. [19]. It seemed that the conclusions drawn by Abdel-Rahman [17] and Li et al. [18] could be reversed. Given the higher heterogeneity, we observed these 15 studies and hypothesized that differences in therapeutic regimens (anti-PD-1/PD-L1 monotherapy/combined chemotherapy) of the experimental arms might be the underlying cause of heterogeneity.

Therefore, subgroup analyses were performed according to therapeutic regimens. The findings were surprising and situations turned to be in two different directions. In monotherapy groups, among non-smokers, no significantly prolonged survival was found as opposed to controls (HR, 0.94, *P* = 0.304), while smokers did significantly benefit from these agents (HR, 0.79, *P* < 0.01). The total groups of monotherapy covered three types of carcinomas closely related with smoking (NSCLC, HNSCC, UC); then, further analysis was performed to define the benefit in different carcinomas. Two separate results were highly consistent with the total results. These real-world outcomes suggested that there must be some underlying mechanism, so we attempted to explain the outcomes with the help of numerous studies.

The tendency of smokers benefiting from anti-PD-1/PD-L1 therapy has been observed in the initial clinical study of durvalumab, which found that smokers had a better response trend to MPDL3280A (durvalumab’s pre-market name) [33]. Since then, to clarify the relationship between smoking status and efficacy of anti-PD-1/PD-L1 therapy, numerous studies emerged. Rizvi et al. [34] found that smoking increased TMB, especially non-synonymous mutations, which further improved the efficacy of anti-PD-1/PD-L1 therapy. The implication is that smoking history is a surrogate marker of tumor mutation and neoantigen burden, and these, in turn, are surrogates for downstream common denominators that ultimately lead to immune recognition of cancer and activation of effective cancer rejection [35]. The relationship between smoking and PD-L1 expression has not been observed in the previous studies [36–38]. However, it has been confirmed recently. Kerdidani et al. [39] tracked the entire process from smoking to emphysema to lung cancer and found that dendritic cells (DCs) exposed to emphysema tumor microenvironment will upregulate PD-L1/IDO expression through oxidative stress-dependent mechanism, mediating immune tolerance, and tumor escape. Zhao et al. [40] demonstrated that melanomas generate a site of immune privilege by driving DCs fatty acid oxidation via a Wnt5a-b-catenin-PPAR-γ signaling pathway that culminates in the induction of IDO enzyme activity, and this study also showed that inhibiting this pathway reverses DCs tolerization and enhances anti-PD-1 antibody efficacy in a transgenic model of melanoma. In the hypoxic environment, Marti et al. [41] found that Vascular endothelial growth factor (VEGF) increases the expression and activity of IDO in DCs, which has a suppressive effect on Ag-specific and mitogen-stimulated lymphocyte proliferation. The tight correlation existing between immune-infiltrate, angiogenesis and cancer progression and dissemination to distant sites and to nodal compartments is now being explored further [42]. Based on the toxicology of tobacco, Wang et al. [43] reported that cigarette smoke and the carcinogen benzopyrene (BaP) induced PD-L1 expression on lung epithelial cells in vitro and in vivo, which was mediated by the aryl hydrocarbon receptor (AhR). Anti-PD-L1 antibody or deficiency in AhR significantly suppresses BaP-induced lung cancer. Much more clinically, by means of multivariate analysis, Ng et al. [35] found that when the level of PD-L1 ≥ 1%, smoking status was the only significant predictor. Thus, they confirmed that smoking status may be the most important and easily available single predictor of efficacy of anti-PD-1/PD-L1 therapy among the relevant clinical characteristics of NSCLC patients with carcinogenic drive. In addition, the relationship between smoking and the efficacy of anti-PD-1/PD-L1 therapy may also be related to the status of tumor-infiltrating lymphocytes (TILs) [44] and other immune modulators such as B7-H3 (CD276) [45]. In frame of this thinking, the concept of cytokine and bystander microenvironmental cells and precursors could be a bit better explained. Some important insights about tumor milieu role in mediating cancer progression are particularly worth concern in both solid and hematologic tumors. In this regard, hematologic tumors may be more representative; Leone et al. [46, 47] found that DCs accumulate in the bone marrow of myeloma patients will protect tumor plasma cells from CD8^+^ T cell killing, and bone marrow endothelial cells (ECs) can sustain a tumor-specific CD8+ T cell subset with suppressive function in myeloma patients. These significant findings implicated that the intimate interaction between endothelial cells, tumor cells and CD8^+^ T cells created a permissive immune microenvironment that allows undisturbed cancer proliferation.

We should mention here that our study suggested that there may be some crosstalk between the smoking status and HPV infection in patients with HNSCC. Therefore, we cannot predict the efficacy of anti-PD-1/PD-L1 monotherapy by smoking status. Generally, smoking is associated with HPV negative, while non-smoking tends to be HPV positive in relation to HNSCC (oral squamous cell carcinoma mainly) [48]. Previous studies have found that the presence of virus-related antigen provides an advantage; patients who are HPV positive are more likely to benefit from anti-PD-1/PD-L1 therapy than patients with negative ones [12]. In two RCTs we included, although the correspondence between smoking status and HPV infection was consistent, the study by Ferris et al. [4] supposed the previous ones, while the study by Cohen et al. [8] was the opposite. Maybe the inherent crosstalk or benefit bias led to the divergence.

People are always willing to narrate the stories about the smokers’ benefit in anti-PD-1/PD-L1 therapy, tending to forget the adverse outcomes of standard treatments (e.g., chemotherapy). As Singal et al. [49] reported, smokers may benefit from anti-PD-1/PD-L1 therapy, but non-smokers still have longer OS than smokers. In previous studies, the adverse outcomes of smokers in chemotherapy have been confirmed. The study conducted by Igawa et al. [50] showed that smoking history was a favorable predictor of efficacy of pemetrexed monotherapy in NSCLC, and long-term smoking history is associated with poor efficacy. After treatment with cisplatin, adverse outcomes of patients with testicular cancer were associated with smoking history [51]. Among patients receiving adjuvant chemotherapy, smoking reduced progression-free survival and overall survival in ovarian cancer [52]. The similar poor prognosis has also been confirmed in HNSCC [53]. Mechanically, Ye et al. [54] recently found that smoking increased the expression of the TM4SF1 gene, which promotes NSCLC proliferation, invasion, and chemo-resistance through regulation of the DDR1/Akt/ERK-mTOR axis. Therefore, poor prognosis in control arms may play a lateral role in highlighting the efficacy of anti-PD-1/PD-L1 therapy.

As for the combined regimen, the findings are dramatic. In four studies limited to NSCLC, compared to the controls without PD-1/PD-L1 inhibitors, greater benefit was found in the non-smokers’ group (HR_(Non-smoker)_ vs. HR_(Smoker)_ = 0.45 vs. 0.72). Considering that non-smokers cannot benefit from anti-PD-1/PD-L1 monotherapy, these reversal outcomes seemed to suggest something significant (HR_(combined regimen)_ vs. HR_(monotherapy)_ = 0.45 vs. 1.01). The findings have not been systematically described so far. Here, we propose the hypothesis that chemotherapy agents may play a role of a sensitizer in the combined regimen. We speculated that the mechanism was related to the expression of PD-L1. A study conducted by Peng et al. [55] supported this hypothesis by showing that, in ovarian cancer, paclitaxel can induce tumor cells to overexpress PD-L1 through the NF-κB pathway, thereby promoting the formation of a tumor immunosuppressive microenvironment. Shin et al. [56] demonstrated that platinum-based chemotherapy can improve the expression level of PD-L1 in tumor cells of NSCLC patients. Thus, in this situation, blocking PD-L1 may achieve significant therapeutic effect, which explained why sequential therapy was still effective, while the addition of ipilimumab turned out to be invalid. Of course, the mechanism of chemotherapy varies from one to another, and to verify the efficacy of a combined regimen, a large number of basic and clinical studies are still required.

Limitations of this meta-analysis should be taken into account. Firstly, it was based on outcomes of trials and not on individual data. Researchers cannot control exposure or outcome assessment and instead must rely on others for accurate recordkeeping. Secondly, most of the trials we included were open-label designs, which may reduce the quality of studies and cause some bias to an extent. Furthermore, the multivariate analysis in original researches can show the impact of several variables; nonetheless, we only examined several cohorts, and some key statistics cannot be measured, and significant biases may affect the selection of controls. Finally, based on the specificity of our study, the disparity in sample size between non-smokers and smokers was also a cause of bias. Although there are many phase II/III trails related to ICIs, extracting the effect size derived from especially subgroup analysis is difficult, leading to deficiency in our sample size.

In conclusion, our meta-analysis suggested that smokers benefit from either anti-PD-1/PD-L1 monotherapy or combined regimen, compared with chemotherapy alone. Considering the cost-effectiveness, smokers were recommended to adopt the monotherapy. For non-smokers, only the combined regimen was feasible in NSCLC. However, the addition of ipilimumab on the basis of chemotherapy turned out to be invalid, compared with chemotherapy alone. Finally, through the analysis of these high-quality RCTs from the real world, we are hoping that our conclusions can be applied effectively in clinical practice.

## Supplementary information

**Additional file 1: Figure S1.** Egger’s funnel plot for publication bias test of the long-term prognostic outcomes of anti-PD-1/PD-L1 therapy, P=0.203.

**Additional file 2: Figure S2.** Sensitivity analysis of the long-term prognostic outcomes of anti-PD-1/PD-L1 therapy (Non-smoker vs. Smoker).

**Additional file 3: Figure S3.** Forest plot of the long-term prognostic outcomes of anti-PD-1/PD-L1 monotherapy in NSCLC (Non-smoker vs. Smoker), P_Non-smoker_=0.921, P_Smoker_<0.001.

**Additional file 4: Figure S4.** Forest plot of the long-term prognostic outcomes of anti-PD-1/PD-L1 monotherapy in other cancer (Non-smoker vs. Smoker), P_Non-smoker_=0.094, P_Smoker_=0.001.

**Additional file 5: Figure S5.** Forest plot of the long-term prognostic outcomes of anti-CTLA-4 therapy (Light smoker vs.Heavy smoker), P_Non-smoker_=0.670, P_Smoker_=0.820.

**Additional file 6: Table S1.** Quality assessment of studies included.

## Data Availability

The datasets supporting the conclusion of this article are included within the article and its additional files.

## Abbreviations

AhR: Aryl hydrocarbon receptor
CIs: Confidence intervals
DCs: Dendritic cells
HNSCC: Squamous cell carcinoma of head and neck
HRs: Hazard ratios
ICIs: Immune checkpoint inhibitors
IDO: Indoleamine 2,3-dioxygenase
NSCLC: Non-small cell lung cancer
OS: Overall survival
RCT: Randomized controlled clinical trial
TILs: Tumor-infiltrating lymphocytes
TMB: Tumor mutation burden
UC: Urothelial carcinoma
VEGF: vascular endothelial growth factor
