# Supplementary figures and images for: Smokers or non-smokers: who benefits more from immune checkpoint inhibitors in treatment of malignancies? An up-to-date meta-analysis
Source: World J Surg Oncol. 2020 Jan 20;18:15. doi: 10.1186/s12957-020-1792-4 (PMC6971889; doi:10.1186/s12957-020-1792-4)

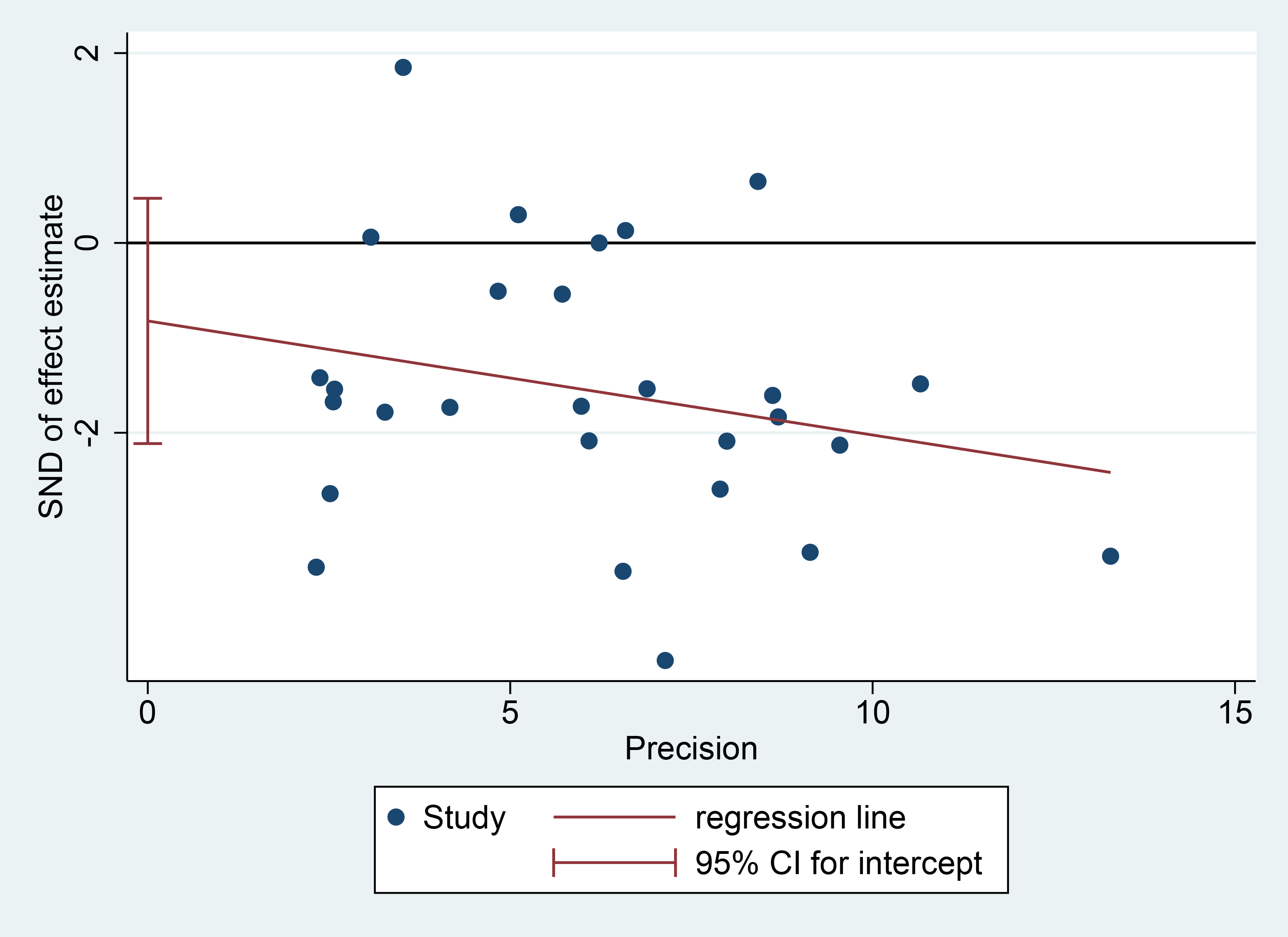

Supplement: Supplementary file 1 — Additional file 1: Figure S1. Egger’s funnel plot for publication bias test of the long-term prognostic outcomes of anti-PD-1/PD-L1 therapy, P=0.203. [file 12957_2020_1792_MOESM1_ESM.tif]

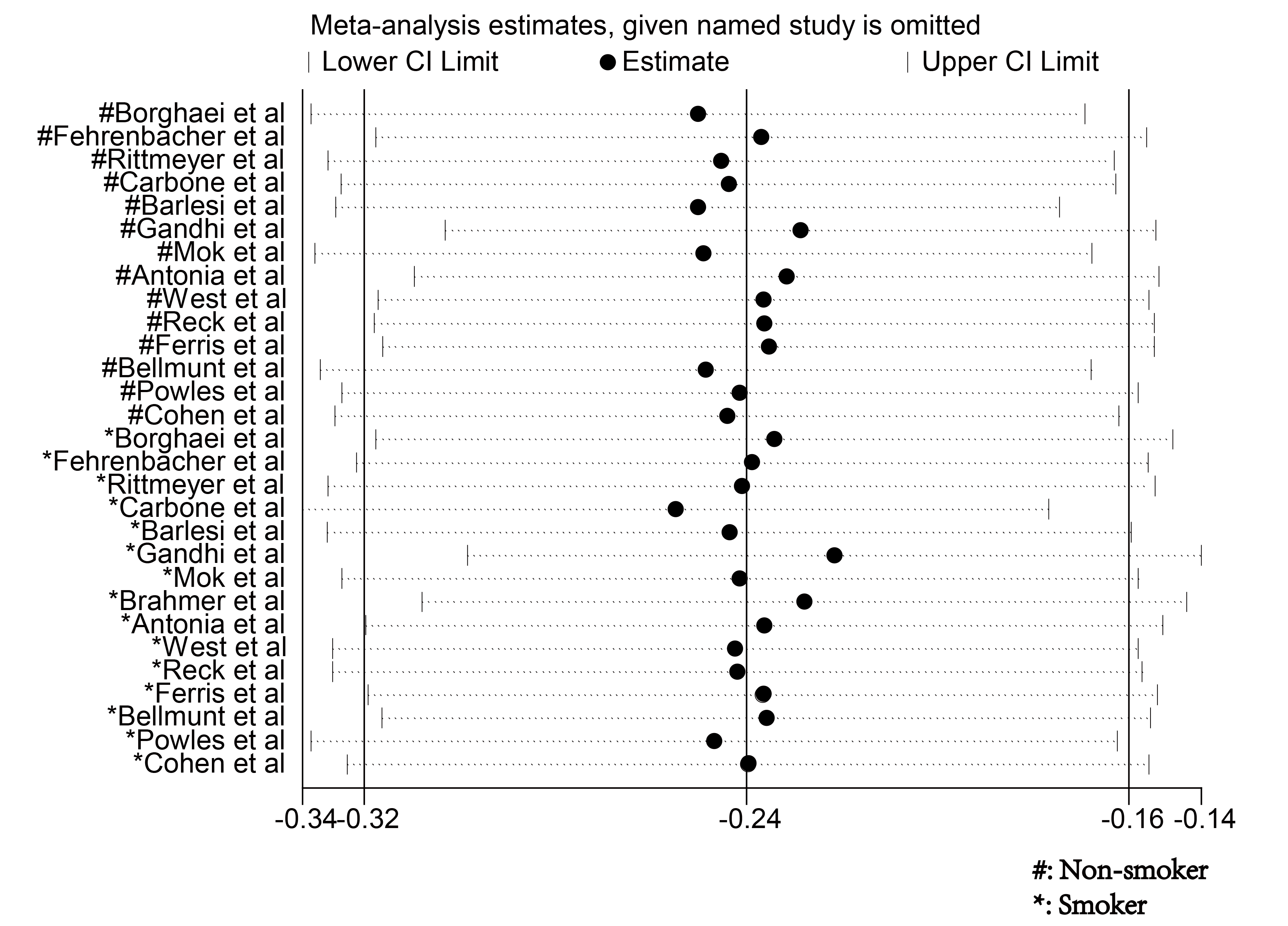

Supplement: Supplementary file 2 — Additional file 2: Figure S2. Sensitivity analysis of the long-term prognostic outcomes of anti-PD-1/PD-L1 therapy (Non-smoker vs. Smoker). [file 12957_2020_1792_MOESM2_ESM.tif]

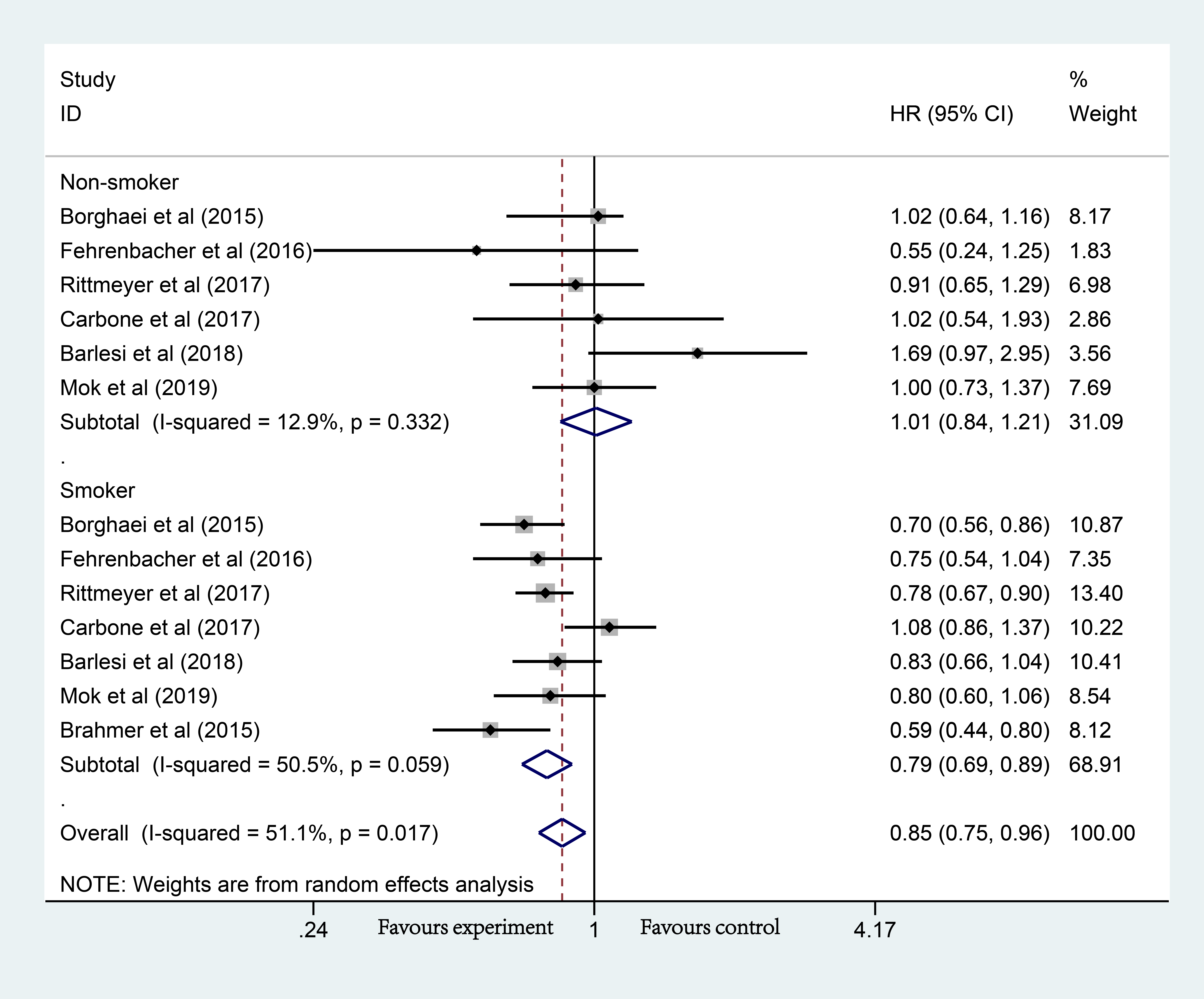

Supplement: Supplementary file 3 — Additional file 3: Figure S3. Forest plot of the long-term prognostic outcomes of anti-PD-1/PD-L1 monotherapy in NSCLC (Non-smoker vs. Smoker), PNon-smoker=0.921, PSmoker<0.001. [file 12957_2020_1792_MOESM3_ESM.tif]

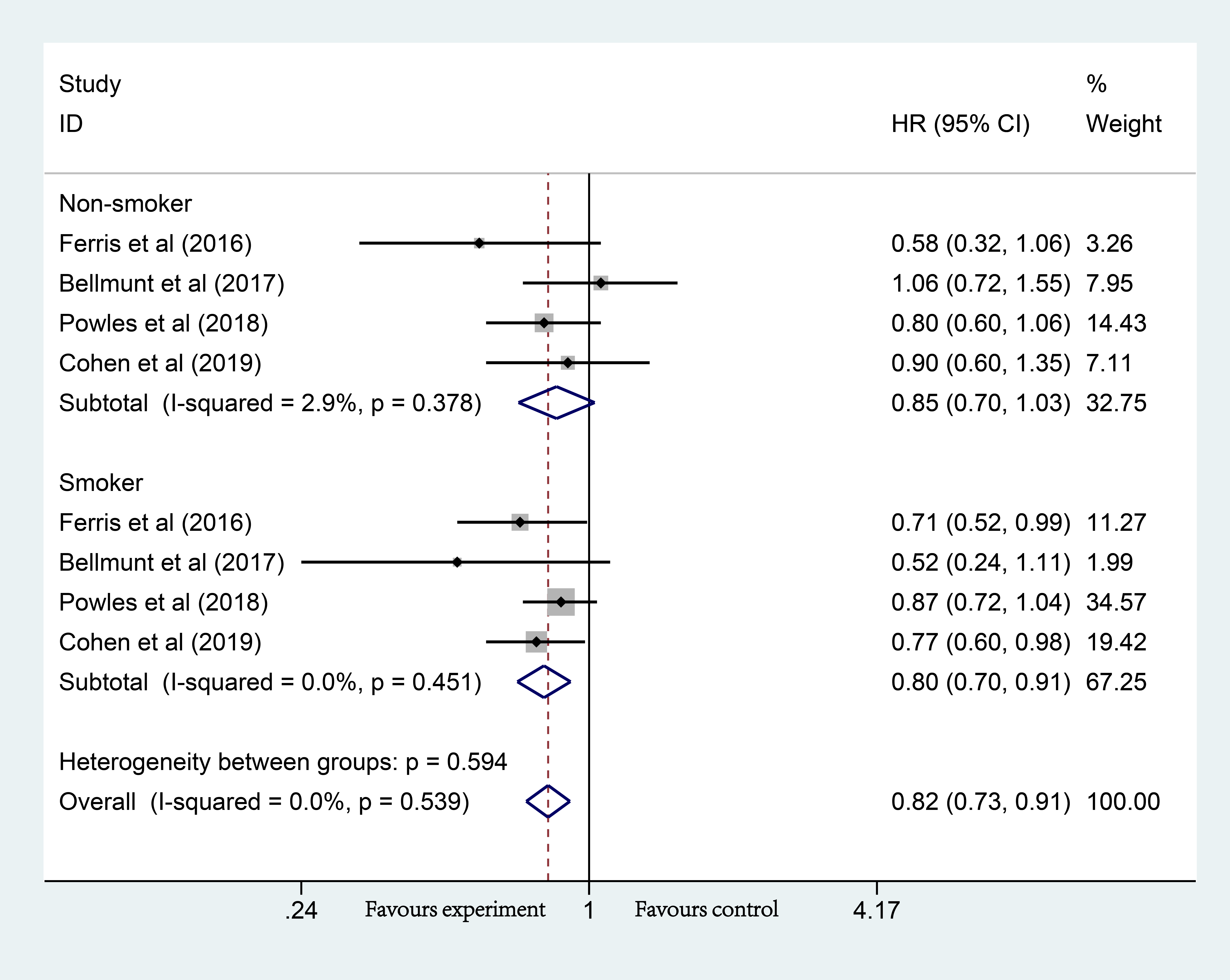

Supplement: Supplementary file 4 — Additional file 4: Figure S4. Forest plot of the long-term prognostic outcomes of anti-PD-1/PD-L1 monotherapy in other cancer (Non-smoker vs. Smoker), PNon-smoker=0.094, PSmoker=0.001. [file 12957_2020_1792_MOESM4_ESM.tif]

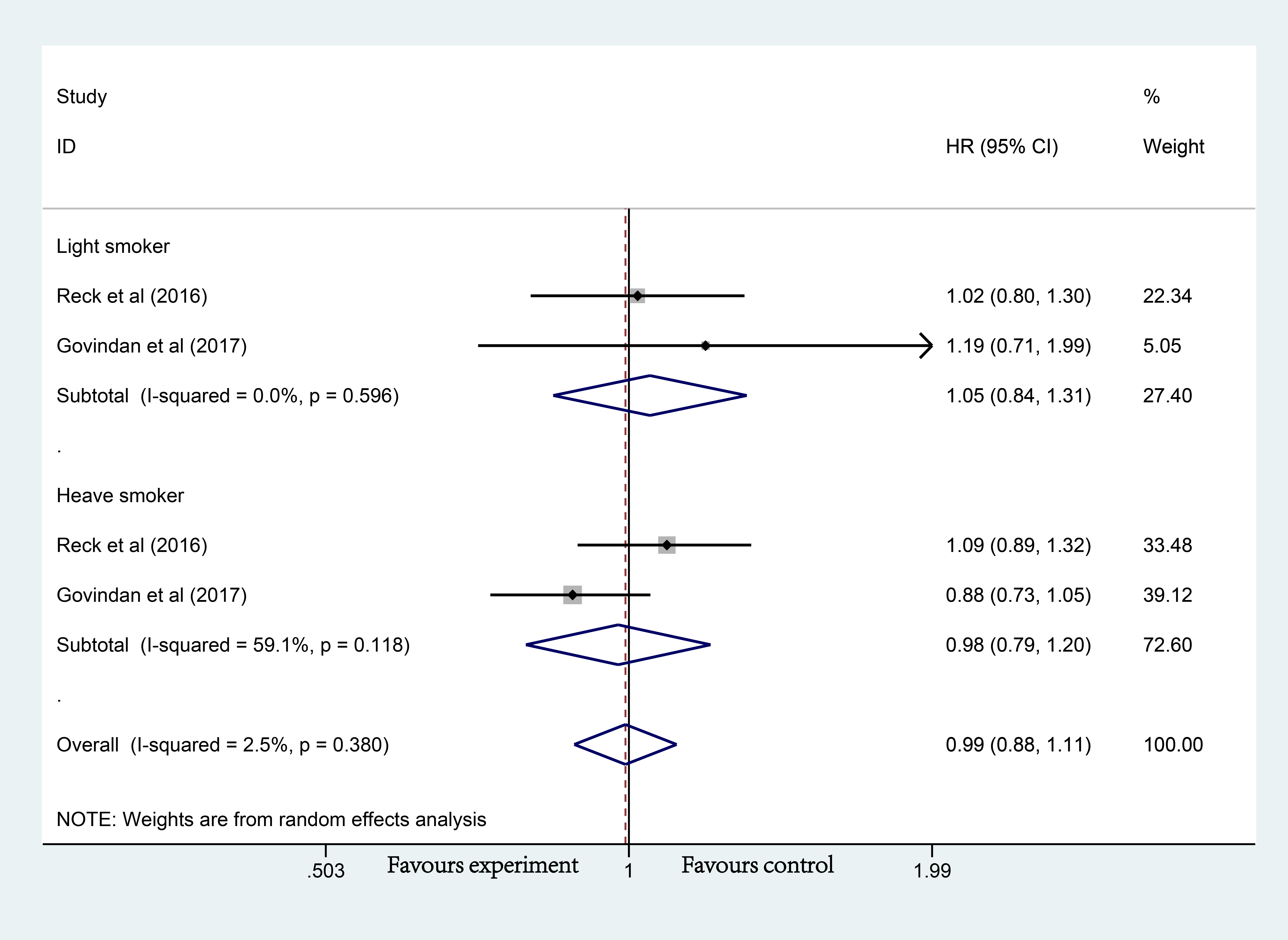

Supplement: Supplementary file 5 — Additional file 5: Figure S5. Forest plot of the long-term prognostic outcomes of anti-CTLA-4 therapy (Light smoker vs.Heavy smoker), PNon-smoker=0.670, PSmoker=0.820. [file 12957_2020_1792_MOESM5_ESM.tif]
