# Supplementary material for: Smokers or non-smokers: who benefits more from immune checkpoint inhibitors in treatment of malignancies? An up-to-date meta-analysis
Source: World J Surg Oncol. 2020 Jan 20;18:15. doi: 10.1186/s12957-020-1792-4 (PMC6971889; doi:10.1186/s12957-020-1792-4)
Supplement: Supplementary file 6 — Additional file 6: Table S1. Quality assessment of studies included. [file 12957_2020_1792_MOESM6_ESM.doc]

**Table S1** Quality assessment of studies included.

| Author, year  Study (RCT) | Sequence  generation | Allocation  concealment | Blinding | Incomplete  outcome data | Selective  outcome reporting | Free of  other bias |
| --- | --- | --- | --- | --- | --- | --- |
| Borghaei et al, 2015 | low risk | low risk | high risk | low risk | low risk | low risk |
| Brahmer et al, 2015 | low risk | low risk | high risk | low risk | low risk | low risk |
| Fehrenbacher et al, 2016 | low risk | unclear risk | high risk | low risk | low risk | low risk |
| Rittmeyer et al, 2017 | low risk | low risk | high risk | low risk | low risk | low risk |
| Carbone et al, 2017 | low risk | low risk | high risk | low risk | low risk | low risk |
| Barlesi et al, 2018 | low risk | low risk | high risk | low risk | low risk | low risk |
| Mok et al, 2019 | low risk | low risk | high risk | low risk | low risk | low risk |
| Gandhi et al, 2018 | low risk | low risk | low risk | low risk | low risk | low risk |
| Antonia et al, 2018 | low risk | low risk | low risk | low risk | low risk | low risk |
| West et al, 2019 | low risk | low risk | high risk | low risk | low risk | low risk |
| Reck et al, 2019 | low risk | low risk | high risk | low risk | low risk | low risk |
| Bellmunt et al, 2017 | low risk | low risk | high risk | low risk | low risk | low risk |
| Powles et al, 2018 | low risk | low risk | high risk | low risk | low risk | low risk |
| Ferris et al, 2016 | low risk | low risk | high risk | low risk | low risk | low risk |
| Cohen et al, 2019 | low risk | low risk | high risk | low risk | low risk | low risk |
| Reck et al, 2016 | low risk | low risk | low risk | low risk | low risk | low risk |
| Govindan et al, 2017 | low risk | low risk | low risk | low risk | low risk | low risk |

The RCTs were assessed by the Cochrane Collaboration’s tool. Risk of bias was assessed as “low risk”, “high risk” or “unclear risk”.
